# Regulation of (Pro)Renin Receptor in Renin-Positive Smooth Muscle Cells of Kidney Arterioles in Rats with STZ-Induced Diabetes

**DOI:** 10.1155/2019/6971928

**Published:** 2019-03-28

**Authors:** Zsolt Razga, Gabor Kovacs, Nikolett Bódi, Petra Talapka, Mária Bagyánszki

**Affiliations:** ^1^Department of Pathology, University of Szeged, Szeged, Hungary; ^2^Department of Pediatrics, University of Szeged, Szeged, Hungary; ^3^Department of Physiology, Anatomy, and Neuroscience, University of Szeged, Szeged, Hungary

## Abstract

*Objective. *The nephron (pro)renin receptor may play a pathophysiological role in renal disorders in hypertension or diabetes. The aim of this study was to determine the relationship of (pro)renin receptors and transdifferentiation between the renin-negative and renin-positive SMCs in the afferent arteriole by estimating the distribution of (pro)renin receptors in renin-positive and renin-negative SMCs of the afferent arteriole of kidneys in normal and streptozotocin- (STZ-) induced diabetic rats. Therefore in diabetes the renin granulation of afferent arterioles is different as in normal, the diabetes model for finding the differences to normal in distribution of (pro)renin receptors of afferent arterioles was used.* Method.* To estimate the number of (pro)renin receptors in arteriolar SMCs a special protocol of immunohistochemistry to stereology was followed.* Results.* Our results showed that on the surface of renin-positive SMCs the number of (pro)renin receptors was upregulated, while in the cytoplasm of SMCs there was downregulation in comparison to renin-negative SMCs. There is a significant difference between the number of (pro)renin receptors on the surface and in the cytoplasm of renin-positive SMCs in normal rats. These differences in the number of (pro)renin receptors were not present in rats with STZ-induced diabetes. Any other differences in the number of (pro)renin receptors between the STZ-induced diabetic and normal rats were not detected. The tissue level of angiotensin II did not change in the kidneys of STZ-induced diabetic rats.* Conclusion.* The distribution of (pro)renin receptors in afferent arteriolar SMCs is related to renin granulation of SMCs, but independent of angiotensin II plasma or tissue levels in the kidney.

## 1. Introduction

The intrarenal renin-angiotensin system (RAS) controls the glomerular filtration rate through glomerular fluid flow and pressure. Systematically circulated renin is synthesized by renin-positive smooth muscle cells (SMCs) of the afferent arteriole and tubule in normal and diabetic rats [1–3]. Renin synthesis in SMCs of afferent arterioles is related to angiotensin II via the angiotensin II receptors [4–6]. The transdifferentiation between the renin-positive and renin-negative SMCs of afferent arterioles in rat kidney is dependent on the number of AT1(A) receptors that are active in the RAS of SMCs [5, 6], similarly to transdifferentiation between the macula densa and tubular cells [7]. The distribution of angiotensin II receptors (AT1(A) and AT1(B)) in rat kidneys differs significantly between normal and STZ-induced diabetes. These changes were related to the renin positivity of afferent arterioles and independent of the intrarenal levels of angiotensin II in diabetic rats [6].

The receptor binding renin and (pro)renin, termed as the (pro)renin receptor, was cloned in 2002 [8]. The (pro)renin receptor has been localized in glomerular mesangial cells and in vascular smooth muscle cells of renal arteries and coronary arteries. It was also found in glomerular epithelial cells and in distal and collecting tubular cells of the kidney [9]. The (pro)renin receptors are highly expressed in developing mouse kidney. In human neonates, the enhanced expression of renal (pro)renin receptors was also detected. These data support the hypothesis that (pro)renin receptors regulate kidney development [10].

The nephron (pro)renin receptor may play a pathophysiological role in renal disorders in hypertension or diabetes [11]. The function of (pro)renin receptors as potential positive regulators in intrarenal RAS has been shown, although it remains to be determined whether prorenin and renin are the true physiological ligand of the (pro)renin receptor [12].

The aim of this study is to determine the relationship of (pro)renin receptors and renin granulation in SMCs in the afferent arteriole by estimating the distribution of (pro)renin receptors in renin-positive and renin-negative SMCs of the afferent arteriole in kidneys of normal and STZ-induced diabetic rats.

## 2. Materials and Method

The induction of diabetes with streptozotocin (STZ) in rats and the measurement of angiotensin II levels were carried out following a previously described protocol [6].

*Animals with STZ-Induced Diabetes*. STZ (Sigma, USA) was administered intraperitoneally at a dose of 60 mg/kg to adult male Wistar rats (300-400 g; n=5). After 48 h, blood sugar levels were measured; animals in which the blood sugar level exceeded 18 mM were regarded as diabetic. The blood sugar level and the weight of the animals were followed over a period of 10 weeks, after which the rats were killed.

*The Measurement of Angiotensin II Levels*. Following the streptozotocin treatment, blood samples were added by phlebotomy from the vena cava posterior into Vacutainer EDTA tubes, while after laparotomy the kidneys were excised and purified from adipose tissue. The high-sensitivity Uscn Life Science Inc. ELISA kit (Cat.no.US-E90005Ra) was used to determine the level of angiotensin II in blood and kidney tissue samples. The tissue and blood were prepared in strict accordance with instructions in the user manual of the kit. 

*Immunohistochemistry*. For immunohistochemical purposes, the same protocol was performed as described earlier [13]. Sections placed on glass slides were washed in PBS (pH = 7.4) for 10 min and then incubated in normal rabbit serum 1:100 (EMS, USA) in PBS (pH = 7.4) for 20 min. The primary antibody against the renin receptor (K-19; sc55025, Santa Cruz Biotechnology, INC; USA) was diluted 1:50 in PBS (pH = 7.4) and left overnight at 4°C in a wetting chamber. The sections were subsequently washed three times for 5 min each in PBS (pH = 7.4). 0.8-nm gold-conjugated rabbit-anti-goat IgG (Aurion Immunogold; EMS, USA) was diluted 1:100 in PBS (pH = 7.4), allowed to stand for 2 h and then washed three times for 5 min each in PBS (pH = 7.4). The sections were fixed with 3% glutaraldehyde in PBS (pH = 7.4) and washed with distilled water for 5 min, followed by silver enhancement with Danscher solution (Aurion R-Gent SE-EM; EMS, USA) for 75 min at 25°C, resulting in a particle size around 30 nm. Finally, the sections were washed three times for 5 min each with distilled water.

*Background Noise of the Immunohistochemistry*. The quality of the immunohistochemical signal at the electron microscope level is crucial for unbiased results. For immunohistochemistry, the (pro)renin receptor (K-19; sc55025, Santa Cruz Biotechnology, INC; USA) was used as the primary antibody, where background-less staining was guaranteed by recombinant technology in the preparation of this reagent. The noise of the labeling system was tested without primary antibody; the use of normal rabbit serum for blocking was sufficient for noiseless staining [13].

*Embedding and Sectioning*. The immunostained serial sections were placed on glass slides and dehydrated with an ordinary series of ethanol and acetone and infiltrated for 10 min with epoxy resin (Embed 812; EMS, USA). The sections were then capped with plastic capsules filled with clean resin. The semithin (0.4-*μ*m) and thin (70+/-5-nm; mean+/-SD) sections were prepared with an Ultracut S (Leica) ultramicrotome. The thin sections were stained with uranyl acetate and lead citrate.

*Stereology*. For sampling, the previously described protocol was followed [13]. An afferent arteriole was defined as a preglomerular arteriole from the glomerulus to the first branching. An efferent arteriole was defined as a postglomerular arteriole from the glomerulus to the capillary. Each arteriole was followed on a series of semithin sections, and profiles appearing from the same arteriole were identified [14, 15]. The TEM micrographs were prepared with JEM-1400 plus electron microscope (JEOL, Peabody, MA, USA). The immunohistochemical signals of the renin receptor molecules were counted on the surface and in the cytoplasm of arteriolar SMCs, renin-granulated cells, and endothelial cells and on renin granules of afferent and efferent arterioles. More details can be found in articles by Razga and Nyengaard [4, 5].

*Statistics*. For the comparison of differences between the two groups, Student's t-test was applied.

## 3. Results

### 3.1. Diabetic Animals

The STZ treatment was successful: the blood sugar levels of the animals increased 3-fold by the end of 10 weeks (Table 1).

### 3.2. Morphology of Diabetic Kidney

There were not found any secondary pathomorphological changes in glomerulus and increments in renin granulation of arterioles.

### 3.3. The Angiotensin II Levels in the Plasma and the Kidney

There were no significant differences between the angiotensin II levels of the plasma or the kidney in the controls and diabetic rats after the 10-week STZ treatment (Table 2).

### 3.4. Immunohistochemical Signal of Renin Receptors in Arterioles

The immunohistochemical signal of renin was found on the surface and in the cytoplasm of renin-positive and renin-negative SMCs of afferent arterioles. The signal was also present in renin vacuoles and on the surface of arteriolar endothelial cells (Figure 3).

### 3.5. Distribution of Renin Receptors in Arterioles

Our results showed no significant differences in the relative number of (pro)renin receptors in renin-positive and renin-negative SMCs of arterioles between the normal and diabetic rats, excluding the efferent arteriolar renin-negative SMCs (Table 3). The relative number of (pro)renin receptors in renin granules or on the surface of endothelial cells did not show significant differences between normal and diabetic rats (Table 4).

**Figure 1:**
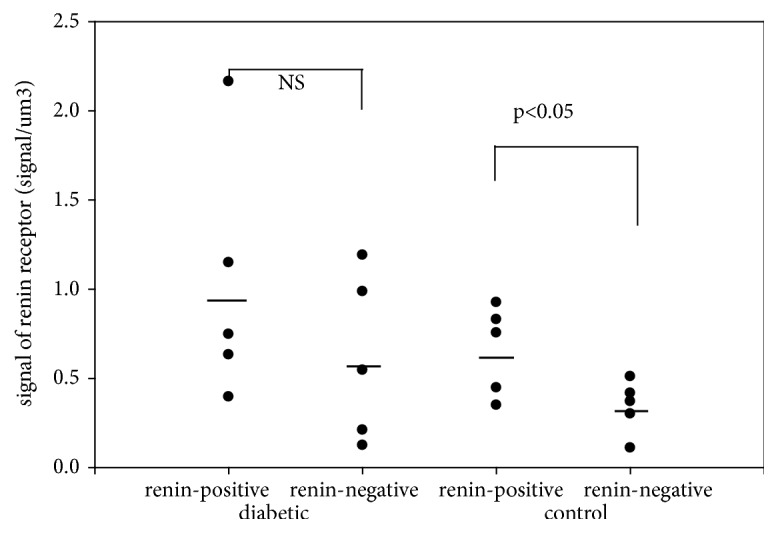
The number of renin receptors on the surface of afferent arteriolar SMCs.

**Figure 2:**
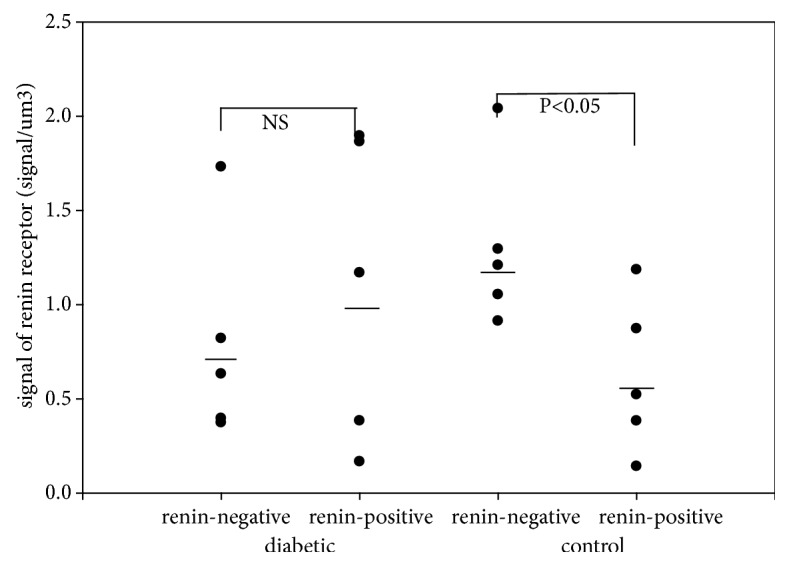
The number of renin receptors in the cytoplasm of afferent arteriolar SMCs.

**Figure 3:**
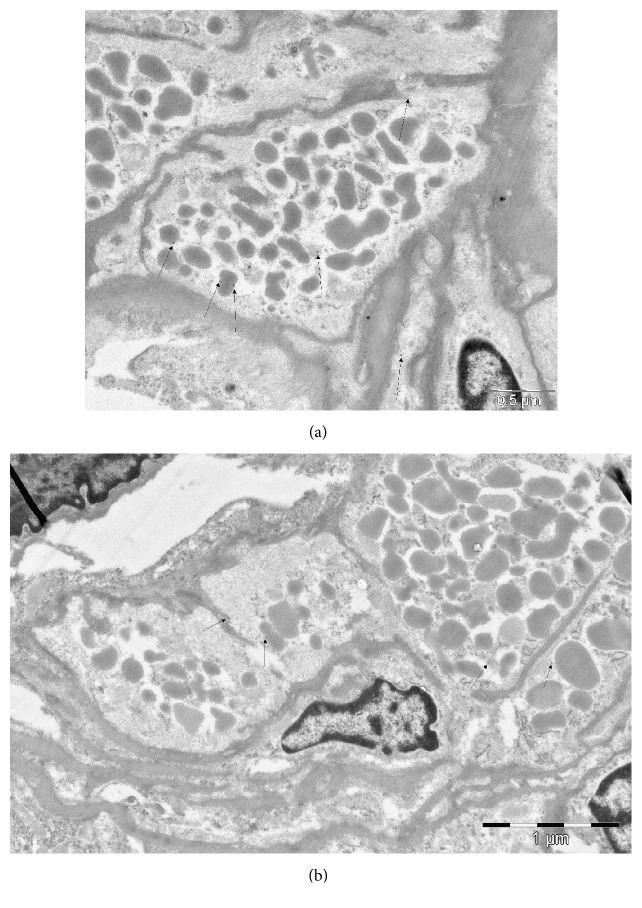
(a-b) Immunohistochemical signal was presented on surface and vacuoles of renin-positive SMCs and in cytoplasm of renin-positive SMCs. About 3000 TEM micrographs were analyzed for estimating the signal of renin receptors.

There were significant differences in the relative number of (pro)renin receptors between the surfaces of renin-negative and renin-positive SMCs of afferent arterioles in kidneys of normal rats (Figure 1). Also, significant differences were found in the relative number of (pro)renin receptors between the cytoplasm of renin-negative and renin-positive SMCs of afferent arterioles in kidneys of normal rats (Figure 2), while significant differences in the relative number of (pro)renin receptors on surfaces and in cytoplasm in STZ-induced diabetic rats were not present (Figures 1 and 2).

Significant differences in the relative number of (pro)renin receptors between the cytoplasm and the surface of renin-positive SMCs were only found in the afferent arterioles of normal rats; in any other SMCs, no significant differences were found between the cytoplasm and the surface (Table 5).

## 4. Discussion

The main issue of this study is whether the (pro)renin receptors could be involved in the intrarenal RAS, that is, whether the renin granulation of SMCs could be changed through the (pro)renin receptors.

In STZ-induced diabetes, renin granulation in the afferent arteriole is higher than normal, while angiotensin II levels in the kidney are also elevated [1, 2, 16, 17]. Earlier investigations clarified this discrepancy, where the heterogeneous regulation of angiotensin II receptors as AT1A and AT1B was presented [6]. The special physiological/pathophysiological state of renin granulation in STZ-induced diabetes has turned attention towards estimating the number (pro)renin receptors in STZ-induced diabetic rats. The distribution of (pro)renin receptors in normal arterioles of the kidney was not examined earlier.

In this study, the estimation of the number of (pro)renin receptors in arteriolar SMCs showed a significant difference between renin-positive and renin-negative SMCs (the surface and cytoplasm) in normal rat kidneys. On the surface of renin-positive SMCs, the number of (pro)renin receptors was upregulated, while in the cytoplasm of SMCs there was downregulation compared to renin-negative SMCs (Figures 1 and 2.). Between the surface and cytoplasm of renin-positive SMCs, the number of (pro)renin receptors shows a significant difference in normal rats (Table 5.). These differences in the number of (pro)renin receptors were not observed in STZ-induced diabetic rats. Any other differences in the number of (pro)renin receptors between the STZ-induced diabetic and normal rats were not detected (Tables 3 and 4.). The plasma or tissue levels of angiotensin II did not change in STZ-induced diabetic rat kidneys (Table 2.).

### 4.1. The Regulation of (Pro)Renin Receptors in Normal Rats

The (pro)renin receptors were localized in glomerular mesangial cells, and in vascular SMCs of renal arteries and coronary arteries. The (pro)renin receptors were also shown by immunohistochemistry to be in glomerular epithelial cells and in distal and collecting tubular cells of the kidney [9]. The (pro)renin receptors have enhanced renal expression in neonates, supporting the hypothesis that the (pro)renin receptor plays an important role in kidney development [10]. The (pro)renin receptors were positioned on the cell surface and in parts of cytoplasm [8, 9]. In this study, the number of (pro)renin receptors was estimated in arteriolar renin-positive and renin-negative SMCs and endothelial cells. The immunohistochemical signal was estimated separately for the surfaces of renin-negative and renin-positive SMCs and endothelial cells and for the cytoplasm of renin-negative and renin-positive SMCs. The signals of immunohistochemistry on membranes of renin vacuoles were also estimated. The (pro)renin receptors were upregulated on the surface of renin-positive SMC and downregulated in the cytoplasm of renin-positive SMCs compared to the renin-negative SMCs. The regulation of (pro)renin receptors in arterioles of normal kidneys was related to renin granulation.

### 4.2. The Regulation of (Pro)Renin Receptors in Diabetic Rats

The expression of (pro)renin receptors in the nephron is increased in angiotensin II-dependent hypertension [11]. In diabetes, (pro)renin expression is also elevated, treatment with a (pro)renin antagonist, such as handle reagent peptide (HRP), reversed the development of diabetic nephropathy [11]. The (pro)renin receptors may regulate renin activity in the distal nephron during angiotensin II-induced hypertension [12], but it has been shown that the (pro)renin receptors can act independently of the renin-angiotensin system. It remains to be determined whether (pro)renin is the true physiologic ligand of (pro)renin receptors [12].

Angiotensin II levels in the kidney are elevated in STZ-induced diabetes [1, 2, 16] and renin granulation is also elevated in afferent arterioles [17]. Our recent demonstration showed that angiotensin II receptor distribution is related to renin granulation in normal and STZ-induced diabetes [5, 6].

In present the study, similar to our earlier study [6], the STZ treatment increased blood sugar levels significantly over 10 weeks (Table 1), while the levels of angiotensin II in the kidney and the plasma were not changed (Table 2). There were not found any secondary pathomorphological changes in glomerulus and increments of renin granulation of arterioles. The (pro)renin receptors in our diabetes model were estimated in this preliminary stage of diabetes. The estimation of (pro)renin receptors in kidney arterioles showed that the (pro)renin receptors are redistributed in diabetes compared to normal. The significant differences in the number of (pro)renin receptors observed between the renin-positive and renin-negative SMCs in normal rat kidneys were not presented in the diabetic sample. These redistributions were independent of angiotensin II plasma or kidney levels.

## 5. Conclusion

The distribution of (pro)renin receptors in afferent arteriolar SMCs is related to renin granulation of SMCs, but independent of the angiotensin II plasma or tissue levels in the kidney. Therefore the (pro)renin receptors could be a possible target to get the pharmacological effects on renin granulation of SMCs in kidney arterioles.

## Figures and Tables

**Table 1:** Changes in blood sugar level and body weight following the STZ diabetic protocol.

			Animal weight (g)	Blood sugar level (mM/l)	
			mean	mean	SD
Control	start	n=4	354	6.7	0.43
	10 weeks	n=4	457	5.53	0.86
Diabetic	start	n=5	360	6.25	0.49
	10 weeks	n=5	318	20.25	9.68

**Table 2:** Angiotensin II levels in the plasma and the kidney (pg/ml).

		Control	Diabetes	
	n	mean (CV)	mean (CV)	
Angiotensin II plasma level (pg/ml)	3	270(0.56)	246(0.29)	NS
Angiotensin II kidney level (pg/ml)	3	666(0.02)	668(0.04)	NS

**Table 3:** The number of renin receptors in renin positive and renin negative SMCs in normal and diabetic rats.

		n	normal	diabetic	p
			mean (CV)		
renin receptor in afferent arterioles cell surface (signals/*μ*m^3^)	Renin-positive SMCs	5	0.66(0.37)	1.02 (0.68)	NS
	Renin-negative SMCs	5	0.34(0.43)	0.61(0.76)	NS
renin receptor in efferent arterioles cell surface (signals/*μ*m3)	Renin-negative SMCs	5	0.42(0.78)	0.99(0.59)	0.047
renin receptor in afferent arterioles cell cytoplasm (signals/*μ*m^3^)	Renin-positive SMCs	5	1.3(0.34)	0.79(0.61)	NS
	Renin-negative SMCs	5	0.62(0.66)	1.09(0.81)	NS

**Table 4:** The number of renin receptors in renin granules and on endothelial surfaces in normal and diabetic rats.

	n	normal	diabetic	p
		mean (CV)		
renin receptor in renin granule of afferent arterioles (signals/*μ*m^3^)	5	1.47(0.83)	0.79(0.61)	NS
renin receptor in endothelia cell surface of afferent arterioles (signals/*μ*m3)	5	0.22(0.71)	0.44(0.53)	NS

**Table 5:** The number of renin receptors on the surface and in the cytoplasm of renin positive and renin negative SMCs.

		n	surface	cytoplasm	p
			mean (CV)		
renin receptor in afferent arterioles renin-positive SMCs (signals/*μ*m^3^)	normal	5	0.66(0.38)	1.3(0.34)	0.01
	diabetic	5	1.02(0.68)	0.79(0.61)	NS
renin receptor in afferent arterioles renin-negative SMCs (signals/*μ*m^3^)	normal	5	0.34(0.44)	0.62(0.66)	NS
	diabetic	5	0.61(0.77)	1.09(0.81)	NS

## Data Availability

The data used to support the findings of this study are available from the corresponding author upon request.
